# Intensive care at the end of life in patients dying due to non-cancer chronic diseases versus cancer: a nationwide study in Denmark

**DOI:** 10.1186/s13054-015-1124-1

**Published:** 2015-11-24

**Authors:** Thomas Lyngaa, Christian Fynbo Christiansen, Henrik Nielsen, Mette Asbjørn Neergaard, Anders Bonde Jensen, Kristina Grønborg Laut, Søren Paaske Johnsen

**Affiliations:** Department of Clinical Epidemiology, Aarhus University Hospital, Olof Palmes Alle 43-45, 8200 Aarhus, Denmark; Department of Oncology, Aarhus University Hospital, Aarhus, Denmark; Division of Epidemiology & Biostatistics, University of Leeds, Leeds, UK

## Abstract

**Introduction:**

It is unknown to what extent use of palliative care and focus on proactive planning of end-of-life (EOL) care among cancer patients is also reflected by less use of intensive care. We aimed to examine the use of intensive care in the EOL in patients dying as a result of non-cancer diseases compared with patients dying due to cancer.

**Methods:**

We conducted a nationwide follow-up study among 240,757 adults dying as a result of either non-cancer chronic disease or cancer in Denmark between 2005 and 2011. Using the Danish Intensive Care Database, we identified all admissions and treatments in intensive care units (ICU) during the patients’ last 6 months before death. We used prevalence ratios (aPRs) adjusted for age, sex, comorbidity, marital status and residential region to compare the 6-month prevalence of ICU admissions as well as treatment with invasive mechanical ventilation (MV), non-invasive ventilation (NIV), renal replacement therapy (RRT) and inotropes and/or vasopressors. In addition, length of ICU stay and death during ICU admission were compared among non-cancer and cancer patients dying between 2009 and 2011.

**Results:**

Overall 12.3 % of non-cancer patients were admitted to an ICU within their last 6 months of life, compared with 8.7 % of cancer patients. The overall aPR for ICU admission was 2.11 [95 % confidence interval (CI) 1.98–2.24] for non-cancer patients compared with cancer patients and varied widely within the non-cancer patients (patients with dementia, aPR 0.19, 95 % CI 0.17–0.21; patients with chronic obstructive lung disease, aPR 3.19, 95 % CI 2.97–3.41). The overall aPRs for treatment among non-cancer patients compared with cancer patients were 1.40 (95 % CI 1.35–1.46) for MV, 1.62 (95 % CI 1.50–1.76) for NIV, 1.19 (95 % CI 1.07–1.31) for RRT and 1.05 (95 % CI 0.87–1.28) for inotropes and/or vasopressors. No difference in admission length was observed. Non-cancer patients had an increased risk of dying in an ICU (aPR 1.23, 95 % CI 0.99–1.54) compared with cancer patients.

**Conclusions:**

Overall, patients dying as a result of non-cancer diseases were twice as likely to be admitted to ICUs at the EOL as patients dying due to cancer. Further studies are warranted to explore whether this difference in use of intensive care reflects an unmet need of palliative care, poor communication about the EOL or lack of prognostic tools for terminally ill non-cancer patients.

**Electronic supplementary material:**

The online version of this article (doi:10.1186/s13054-015-1124-1) contains supplementary material, which is available to authorized users.

## Introduction

Chronic disease remains the dominant cause of death globally, with cancer being the leading cause [1], followed by heart, cerebrovascular and lung diseases [2]. Patients with chronic disease require increased care, which may include intensive care, at the end of life (EOL) [3], defined as the last 6 months before death. Ageing populations translate to a higher prevalence of chronic diseases [4], and therefore increased spending on EOL care in the coming years is expected. More than one-fourth of all health care costs in the United States are already being incurred during the last year of patients’ lives [5]. The majority (>80 %) of these expenses are for intensive care [6].

Use of palliative treatment is increasing, particularly for cancer patients [7]. However, studies have shown that patients with heart failure experience a burden of symptoms similar to that of patients with advanced cancer [8]. In addition, patients with chronic obstructive pulmonary disease (COPD) were found to receive less palliative care at the EOL than patients with lung cancer, despite having comparable symptoms [9], and were more likely than cancer patients to die in a hospital setting instead of at home [10].

Intensive care may constitute a substantial emotional and physiological burden for both patients and their relatives [11]. Deciding who should be admitted to an intensive care unit (ICU) remains a difficult task; however, a widely accepted consensus is that a considerable prospect of recovery must exist [12]. Meanwhile, ensuring relevant, high-quality care that meets the expectations of patients and their relatives at all stages of illness in a health care system with limited resources is a major challenge [5]. Timely recognition of a non-curative disease stage and open discussions about prognosis and preferences could presumably ease this task [13, 14]. Existing research on the use of intensive care during the EOL has been focused on few diagnoses [9] or variation over time [10] or has not actually compared the differences between patients or diagnostic groups [3, 6, 15], leaving a requirement for more data on variation in use of intensive care to provide a better understanding of disease patterns and thereby support clinicians in the rational use of intensive care for patients with cancer and other chronic diseases.

The differences in care patterns between non-cancer and cancer patients are of particular interest because an increased level of care at the EOL does not seem to be associated with better survival, higher functional status or improved quality of life [16, 17], nor has it been shown to be aligned with patients’ preferences for treatment and place of death, leaving many patients without the care they wish for in their final months of life [18, 19].

The nationwide clinical databases and population-based medical registries in Denmark provide a unique opportunity to investigate the use of intensive care at the EOL in a setting with equal and universal access to health care. The aim of this study was to examine the use of intensive care and death in the ICU at the EOL and compare patients who died as a result of non-cancer chronic diseases with those who died due to cancer.

## Methods

### Study design and setting

We conducted this nationwide follow-up study in Denmark, a country with a population of approximately 5.6 million. The health care system of Denmark is financed through federal taxes and provides equal, universal access to hospital care, including intensive care, for all citizens. More than 98 % of Danish citizens are registered with a general practitioner (GP). GPs act as gatekeepers for access to specialists and hospital treatment. Interdisciplinary palliative specialist teams are available for referral from GPs and hospital specialists [20]. Denmark encompasses 49 ICUs (2011). Unambiguous individual-level linkage between population-based registries was performed using the unique civil registration number assigned to each Danish citizen at birth and to residents upon immigration [21].

### Study population

The Danish Registry of Causes of Death was used to identify the underlying cause of death for all decedents from the age of 18 years who died between 1 January 2005 and 31 December 2011. The Danish Registry of Causes of Death contains data on all decedents since 1970, and data entry is mandatory by law. Data include, among others, civil registration number; date of death; manner of death; and cause of death, both immediate and underlying, coded according to the Danish version of the International Classification of Diseases, Tenth Revision [22]. We grouped the underlying causes of death into two groups: cancer or non-cancer (diabetes, dementia, ischaemic heart disease, congestive heart failure, cerebrovascular disease, COPD and chronic liver disease) (Additional file 1). These eight causes of death were the most common in Denmark in the 2005–2011 period [23]. The remaining causes of death were grouped as ‘other’ and comprised 144,010 individuals (37.4 %) who were excluded from the analyses. Likewise, 48 patients (<0.1 %) with missing information on residential region were excluded.

### Intensive care

Data on ICU admission within the last 6 months before death were identified through the Danish Intensive Care Database (DID). The DID is a clinical database established for nationwide quality monitoring and holds data from 2005 and onwards for patients admitted to any ICU in Denmark. Data entry is mandatory by law. The positive predictive value of data on ICU admissions in the DID has been found to be between 87.2 % and 98.7 % [24, 25]. Data include, among others, information on admission date; discharge status, including death in an ICU; invasive mechanical ventilation (MV); non-invasive ventilation (NIV); inotrope and/or vasopressor therapy; and renal replacement therapy (RRT) [26].

### Comorbidities

We obtained data on comorbid conditions using diagnoses from hospital admissions and outpatient clinical visits recorded in the Danish National Registry of Patients in up to the 10 years preceding death. We assessed comorbidity level by means of the Charlson comorbidity index (CCI) [27]. This scoring system assigns between 1 and 6 points to each of the 19 conditions. The standard CCI was calculated from the sum of weights for the 19 diseases [27]. We further modified the CCI by deducting points for the underlying cause of death if it was also present as comorbidity. We then calculated modified scores summing the weights for the other comorbid condition (Additional file 2). This was done to avoid including diseases in the analyses as both comorbid conditions and causes of death. Patients with a modified CCI score ≥1 were categorised as ‘any comorbidity’, and patients with a modified CCI of 0 were categorised as ‘no comorbidity’.

### Statistical analyses

The period prevalence of admission to ICU within 6 months before death for patients dying as a result of non-cancer chronic diseases and for patients dying due to cancer was calculated and compared by adjusted prevalence ratios (aPRs), which were estimated using multivariable binomial regression adjusted for age, sex, comorbidity and marital status. In all adjusted analyses, we accounted for potential clustering by residential region. We repeated the analyses stratified by age groups and sex. Next, we calculated the prevalence proportions of patients treated with invasive MV, NIV, RRT and inotropes and/or vasopressors among patients admitted to an ICU within the last 6 months before death. Analyses were stratified according to age and sex. Aggressiveness of treatment was defined as either ‘full organ supportive treatment’ (i.e., the patient received respiratory support by MV and/or NIV, vasopressor and/or inotropes and RRT during ICU admissions in the last 6 months of life) or as ‘partial organ supportive treatment’ (i.e., the patient received treatment in two or less of the three treatment modalities assessed) (Additional file 3). We calculated the median length of ICU stay along with the interquartile range (IQR). Due to availability of data in the DID, we restricted this analysis to the years 2009–2011. Finally, we calculated the proportion of deaths occurring in an ICU. This analysis was also restricted to the years 2009–2011 due to the availability of these data in the DID. The proportions of deaths in ICUs were compared for the non-cancer and cancer patients using multivariable binomial regression adjusted for age, sex, any comorbidity and marital status.

All statistical analyses were performed using Stata software (Stata/IC version 13.1; StataCorp, College Station, TX, USA). In accordance with National Committee on Health Research Ethics guidelines, non-interventional studies do not require approval from ethics committees in Denmark. The study was approved by the Danish Data Protection Agency (record numbers 2009-41-3987 and 2014-41-3658).

## Results

### Descriptive data

**Fig. 1:**
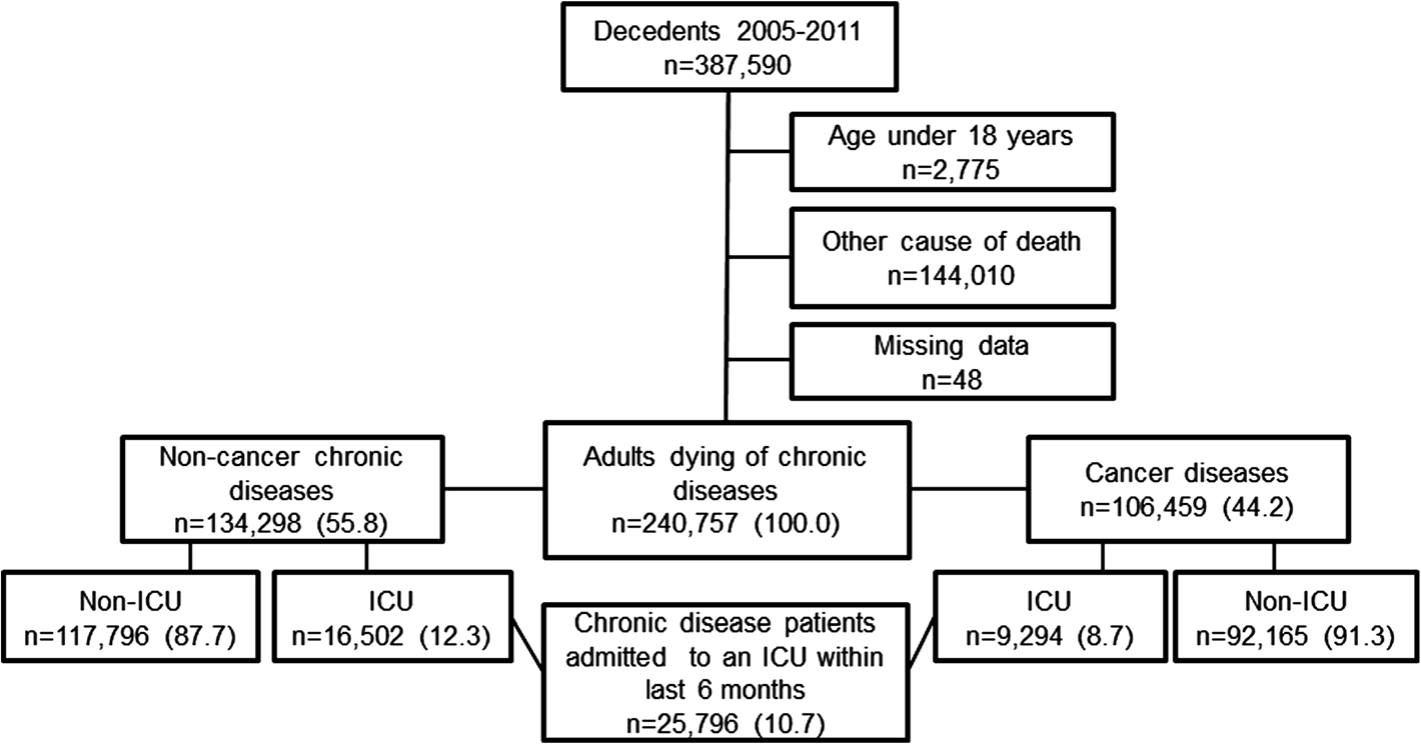
Flowchart depicting inclusion criteria of study population and grouping of cancer and non-cancer patients [number (%)]. *ICU* intensive care unit

We included a total of 240,757 adult decedents during the 7-year study period (Fig. 1). Among these individuals, 134,298 (55.8 %) died as a result of the included non-cancer diseases and 106,459 (44.2 %) died due to cancer. The median ages were 82 years for non-cancer patients and 74 years for cancer patients. Women comprised 52.6 % of the non-cancer group and 48.7 % of the cancer group (Table 1).

**Table 1:** Characteristics of decedents between 2005 and 2011, stratified by underlying cause of death as either cancer or chronic non-cancer disease

	Chronic non-cancer disease, n (% of all non-cancer patients)	Cancer, n (% of all cancer patients)	Total study population, n (% of all decedents)
Overall number (%)	134,298 (100.0 %)	106,459 (100.0 %)	240,757 (100.0 %)
Median age [IQR], yr	82 [74–89]	74 [65–82]	79 [69–86]
Age group, stratified by sex			
18–49 yr, female	922 (0.7 %)	2483 (2.3 %)	3405 (1.4 %)
18–49 yr, male	2121 (1.6 %)	1973 (1.9 %)	4094 (1.7 %)
50–59 yr, female	2232 (1.7 %)	5742 (5.4 %)	7974 (3.3 %)
50–59 yr, male	4650 (3.5 %)	5809 (5.5 %)	10,459 (4.3 %)
60–69 yr, female	5309 (4.0 %)	11,772 (11.1 %)	17,081 (7.1 %)
60–69 yr, male	9745 (7.3 %)	14,043 (13.2 %)	23,788 (9.9 %)
70–79 yr, female	13,789 (10.3 %)	14,939 (14.0 %)	28,728 (11.9 %)
70–79 yr, male	16,854 (12.5 %)	17,650 (16.6 %)	34,504 (14.3 %)
80–89 yr, female	29,629 (22.1 %)	13,396 (12.6 %)	43,025 (17.9 %)
80–89 yr, male	22,991 (17.1 %)	13,042 (12.3 %)	36,033 (15.0 %)
90+ yr, female	18,818 (14.0 %)	3469 (3.3 %)	22,287 (9.3 %)
90+ yr, male	7238 (5.4 %)	2141 (2.0 %)	9379 (3.9 %)
Sex			
Female	70,699 (52.6 %)	51,801 (48.7 %)	122,500 (50.9 %)
Male	63,599 (47.4 %)	54,658 (51.3 %)	118,257 (49.1 %)
Marital status			
Married	42,635 (31.7 %)	53,771 (50.5 %)	96,406 (40.0 %)
Unmarried	17,158 (12.8 %)	14,045 (13.2 %)	31,203 (13.0 %)
Divorced	12,512 (9.3 %)	8494 (8.0 %)	21,006 (8.7 %)
Widowed	61,993 (46.2 %)	30,149 (28.3 %)	92,142 (38.3 %)
Geographical region of residence			
North Denmark Region	15,715 (11.7 %)	11,931 (11.2 %)	27,646 (11.5 %)
Central Denmark Region	27,689 (20.6 %)	22,428 (21.1 %)	50,117 (20.8 %)
Region of Southern Denmark	31,080 (23.1 %)	23,669 (22.7 %)	54,749 (22.7 %)
Capital Region of Denmark	38,138 (28.4 %)	30,680 (28.6 %)	68,818 (28.6 %)
Region Zealand	21,676 (16.1 %)	17,751 (16.7 %)	39,427 (13.4 %)
Cause of death			
Cancer	–	106,456 (100.0 %)	106,456 (44.2 %)
Non-cancer chronic diseases	134,298 (100.0 %)	–	134,298 (55.8 %)
Diabetes	9150 (6.8 %)	–	9150 (3.8 %)
Dementia	18,298 (13.6 %)	–	18,298 (7.6 %)
Ischaemic heart disease	39,466 (29.4 %)	–	39,466 (16.4 %)
Heart failure	10,779 (8.0 %)	–	10,779 (4.5 %)
Cerebrovascular disease	28,522 (21.2 %)	–	28,522 (11.9 %)
COPD	22,120 (16.5 %)	–	22,120 (9.2 %)
Chronic liver failure	5963 (4.4 %)	–	5963 (2.3 %)
ICU admission^a^			
No	117,796 (87.7 %)	97,165 (91.3 %)	214,961 (89.3 %)
Yes	16,502 (12.3 %)	9294 (8.7 %)	25,796 (10.7 %)
Comorbidity^b^			
No	48,299 (36.0 %)	32,876 (30.9 %)	81,175 (33.7 %)
Yes	85,999 (64.0 %)	73,583 (69.1 %)	159,582 (66.3 %)

*COPD* chronic obstructive pulmonary disease, *ICU* intensive care unit, *IQR* interquartile range

^a^Any admission to an ICU within the last 6 months before death

^b^Calculated as Charlson comorbidity index diseases, excluding underlying cause of death

### ICU admission

Within the last 6 months before death, 25,796 (10.7 %) of all patients were admitted to an ICU (Table 1), accounting for 12.3 % of the non-cancer patients and 8.7 % of the cancer patients. The overall aPRs for admission to an ICU during the last 6 months before death were 2.11 [95 % confidence interval (CI) 1.98–2.24) among patients dying of non-cancer disease compared with cancer. Compared with cancer patients, those dying of COPD were more likely to be admitted to an ICU during EOL (aPR 3.19 (95 % CI: 2.97–3.41)), while patients dying of dementia were less likely to be admitted to an ICU (aPR 0.19 (95 % CI: 0.17–0.21)) (Table 2).

**Table 2:** ICU admission during the last 6 months before death, by cause of death

	ICU admission		
Causes of death	Admitted to ICU (%)	aPR	95 % CI
Cancer	8.7 %	1.00	Reference
Chronic non-cancer diseases	12.3 %	2.11	1.98–2.24
Diabetes	11.4 %	1.58	1.34–1.86
Dementia	0.7 %	0.19	0.17–0.21
Ischaemic heart disease	10.2 %	1.69	1.52–1.88
Heart failure	9.8 %	1.95	1.86–2.03
Cerebrovascular disease	13.2 %	2.39	2.17–2.63
Chronic obstructive pulmonary disease	31.9 %	3.19	2.98–3.41
Chronic liver failure	27.4 %	2.42	1.94–3.03

*aPR* adjusted prevalence ratio (adjusted for age, sex, marital status, any comorbidity and geographic region), *CI* confidence interval, *ICU* intensive care unit

**Fig. 2:**
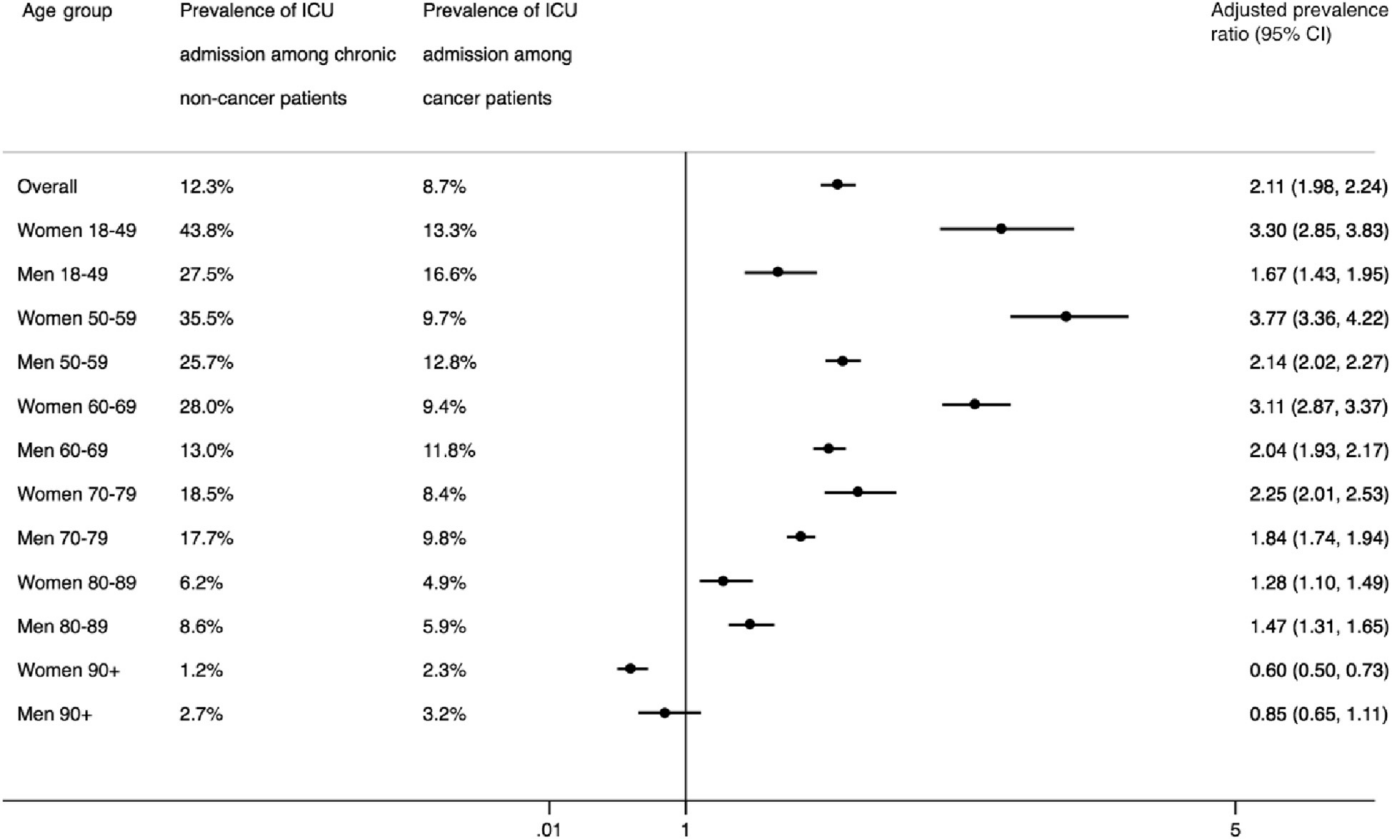
Forest plot of the adjusted prevalence ratios for admission to an intensive care unit (ICU) within the last 6 months before death among patients dying as a result of non-cancer chronic diseases compared with patients dying due to cancer. *CI* confidence interval

Figure 2 shows the prevalence of ICU admission by age group and sex. For both sexes, we found the highest aPRs for the 50–59-year-old age group (aPR women 3.77, 95 % CI 3.36–4.22; aPR men 2.14, 95 % CI 2.02–2.27) when we compared non-cancer patients with cancer patients. The difference between non-cancer and cancer patients progressively declined with age for both sexes, with the age 90+ years group having the lowest aPR estimates (aPR women 0.60, 95 % CI 0.50–0.73; aPR men 0.85, 95 % CI 0.65–1.11) (Fig. 2).

### ICU treatment

Table 3 displays data on the use of ICU-specific treatment modalities. Overall, these modalities were used more often in non-cancer patients than for cancer patients. Of the non-cancer patients, 55.6 % versus 42.2 % of the cancer patients received MV, corresponding to an aPR 1.40 (95 % CI 1.35–1.46). NIV treatment was given to 27.3 % of the non-cancer patients and 16.4 % of the cancer patients (aPR 1.62, 95 % CI 1.50–1.76), ranging from aPRs of 0.51 (95 % CI 0.45–0.58) for patients dying as a result of dementia to 3.40 (95 % CI 3.00–3.85) for patients dying due to COPD. Treatment with inotropes or vasopressors was used in 44.3 % of the non-cancer patients and 38.9 % of the cancer patients, resulting in an overall aPR of 1.19 (95 % CI 1.07–1.31), whereas no difference in use of RRT was observed (aPR 1.05, 95 % CI 0.87–1.28). No patients with dementia were treated with RRT, whereas the highest aPR was found among patients dying as a result of diabetes (aPR 2.27, 95 % CI 1.91–2.69) (Table 3).

**Table 3:** Treatment in the ICU during the last 6 months before death, by cause of death

	Mechanical ventilation			NIV			Inotropes/vasopressors			Dialysis			Full organ supportive treatment		
Causes of death	%	aPR	95 % CI	%	aPR	95 % CI	%	aPR	95 % CI	%	aPR	95 % CI	%	aPR	95 % CI
Cancer	42.2	1.00	Reference	16.4	1.00	Reference	38.9	1.00	Reference	9.7	1.00	Reference	7.5	1.00	Reference
Chronic non-cancer diseases	55.6	1.40	1.35–1.46	27.3	1.62	1.50–1.76	44.3	1.19	1.07–1.31	9.4	1.05	0.87–1.28	6.9	1.02	0.82–1.27
Diabetes	49.7	1.27	1.16–1.39	17.3	1.03	0.94–1.14	48.2	1.31	1.18–1.46	22.2	2.27	1.91–2.69	14.3	1.98	1.60–2.44
Dementia	25.6	0.80	0.68–0.94	11.3	0.64	0.46–0.88	16.5	1.31	1.18–1.46	0	–	–	0	–	–
IHD	59.3	1.57	1.53–1.61	16.4	0.96	0.90–1.03	62.5	0.54	0.39–0.73	14.0	1.60	1.20–2.14	11.0	1.70	1.31–2.20
Heart failure	49.8	1.36	1.22–1.51	27.8	1.64	1.53–1.75	52.2	1.72	1.60–1.86	14.5	1.74	1.33–2.28	10.2	1.66	1.16–2.39
Stroke	69.6	1.66	1.60–1.71	8.2	0.51	0.45–0.58	33.3	1.49	1.32–1.68	3.6	0.40	0.32–0.50	2.64	0.37	0.29–0.48
COPD	43.3	1.11	1.01–1.22	58.5	3.40	3.00–3.85	33.3	0.86	0.72–1.03	5.3	0.62	0.46–0.84	3.8	0.59	0.38–0.91
CLF	61.1	1.30	1.14–1.47	13.4	0.90	0.81–1.01	52.2	0.90	0.79–1.03	12.6	1.19	0.90–1.56	9.4	1.12	0.83–1.52

*aPR* adjusted prevalence ratio (adjusted for age, sex, marital status, any comorbidity and geographic region), *CI* confidence interval, *COPD* Chronic obstructive pulmonary disease, *CLF* Chronic liver failure, *IHD* ischaemic heart disease

When we examined the combination of treatments (Additional file 3), we found that the proportions receiving full organ supportive treatment were 6.9 % among the non-cancer patients and 7.5 % among the cancer patients. No difference between non-cancer patients and cancer patients was observed after controlling for the aforementioned potential confounding (overall aPR 1.02, 95 % CI 0.82–1.27). However, no patients who were dying as a result of dementia received full organ supportive treatment, and the highest aPR was found among patients dying due to diabetes (aPR 1.98, 95 % CI 1.60–2.44) (Table 3).

### Length of ICU stay

The overall median length of stay per ICU admission within the last 6 months before death was 29.5 h for the non-cancer patients (IQR 10.1–87.6). For cancer patients, the corresponding number was 29.7 h (IQR 13.2–94.5) (Table 4).

**Table 4:** Length of ICU admission per admission

	Overall		2009		2010		2011	
	Non-cancer	Cancer	Non-cancer	Cancer	Non-cancer	Cancer	Non-cancer	Cancer
Hours, median (IQR)	29.5 (10.1–87.6)	29.7 (13.2–94.5)	24.5 (8.5–78.7)	24.0 (8.8–90.4)	26.7 (10.1–90.6)	31.5 (13.8–104.1)	32.5 (11.4–89.1)	31.3 (14.8–93.6)
Number (% missing)	4948 (32.4 %)	2844 (30.7 %)	1207 (51.4 %)	597 (54.1 %)	1760 (27.9 %)	1019 (25.3 %)	1981 (17.3 %)	1228 (14.6 %)

### ICU death

The overall proportion of patients dying during ICU admission was 35.5 % among non-cancer patients and 29.2 % for cancer patients. We found an overall increased risk of dying during ICU admission for non-cancer in comparison with cancer patients (aPR 1.23, 95 % CI 0.99–1.54) (data not shown).

## Discussion

In this Danish nationwide study, we found considerably higher use of ICU admission at the EOL among patients dying as a result of non-cancer chronic diseases than among patients dying due to cancer. In comparison with patients dying due to cancer, patients with dementia as the underlying cause of death were unlikely to be admitted to the ICUs and received less treatment, whereas the opposite was the case for patients dying as a result of COPD or diabetes. Whereas there was no overall difference in the prevalence of full organ supportive treatment between non-cancer and cancer patients, patients dying as a result of diabetes or heart disease received full organ supportive treatment almost two times more often than cancer patients did.

The findings of our study are supported by those of a smaller U.S. study [9] in which researchers compared the health care resource use of 1490 patients with COPD at Veteran Affairs medical centres with 459 patients with lung cancer. Those authors found that patients with COPD were twice as likely to be admitted to an ICU in the last 6 months before death as those with lung cancer. In our study, we found that patients dying as a result of COPD were admitted to an ICU three times more often than all patients dying due to cancer. Whereas the sample population in the U.S. study was predominantly elderly white men, our study included both men and women. The difference in case mix between the U.S. study and our study could likely explain the higher admission rate in our study, as our findings suggest that the rates of admission to the ICU were lower among men than among women.

To our knowledge, no previous studies have directly evaluated the risk of dying in an ICU for non-cancer patients compared with cancer patients. One U.S. study measured terminal admissions associated with intensive care among non-federal hospitals in six states as a measure of death during ICU admission [6]. The results of that study were that 22.4 % of patients died in hospital after ICU admission. Another U.S. study assessed prevalence of death among patients using intensive care services during terminal hospitalisation in England and the United States [15]. The investigators in that study found hospital mortality among the patients who were admitted to ICUs in England to be 19.6 % and 7.4 % in the United States. In neither of these studies were distinctions made between non-cancer and cancer causes of death. In our study, we were able to assess the prevalence of death during ICU admissions among all patients dying as a result of non-cancer diseases and compare it with the prevalence of ICU deaths among all patients dying due to cancer. When we adjusted for confounders, we found dying in an ICU to be more likely to be associated with dying as a result of non-cancer diseases than dying due to cancer. Bearing in mind that only about half of cancer patients have their wishes fulfilled regarding place of death [19], this adds to the need for a better understanding of the large variation in care patterns between non-cancer patients and cancer patients at the EOL.

We found substantial variation in health care at the EOL, which is consistent with the previously mentioned studies, indicating that triage may be based more on diagnosis and less likely to be driven by symptoms and prognosis, thus raising a question whether treatment is aligned with patients’ preferences.

A number of limitations should be taken into consideration when interpreting our results. First, we examined intensive care during the EOL using a decedent-only sample. This approach has been criticized, as it artificially removes the uncertainty of prognostication in patients near the EOL [28]. However, with the data available for this study, it was not possible to determine when patients with non-cancer chronic conditions entered the terminal phase, which made a traditional follow-up study among all patients with these conditions difficult. We therefore included all adult decedents who died as a result of the eight specified chronic diseases during the study period. Cause of death was determined as the underlying cause of death derived from the Danish Registry of Causes of Death. The Danish Registry of Causes of Death is practically complete [22]. We based our analyses on the underlying cause of death due to well-defined chronic diseases. However, determining the causes of death—both underlying and immediate—can be difficult. Validation of the Danish Registry of Causes of Death has been performed only for some diseases [29], leaving some uncertainty about classification of the causes of death. This could introduce misclassification that is likely to be independent of ICU admission (i.e., non-differential), which would bias the results towards the null association.

In this study, we aimed to control for confounding by adjusting for a range of known potential confounders; however, unmeasured confounding cannot be ruled out. Of the variables we included in our study, those affecting the estimates most were age, sex and marital status.

In the ELDICUS project [12], a wide array of intensive care experts generally agreed on a range of principles expressed in a large consensus statement regarding the triage of ICU patients. Among these was that there must be a considerable prospect for the patient to recover. If symptoms experienced by non-cancer patients are similar to or worse than symptoms experienced by cancer patients [8, 9, 30], then treatment should vary only by a little. However, difficulties in predicting trajectories for non-cancer chronic diseases are offered as an explanation of the existence of differences in treatment [13, 14, 31]. This difficulty is also reflected in the reduced tendency to recognize these patients as having a terminal prognosis [32]. The descriptive nature of the present study does not allow us to determine whether the observed differences in use of intensive care during the EOL are appropriate; we can only speculate about this. Nonetheless, the difference in ICU use between non-cancer and cancer patients found in this study warrants consideration of whether the current allocation of ICU beds is optimal and how to better accommodate the demands of care for non-cancer patients at the EOL.

## Conclusions

In our study, we found that patients dying as a result of non-cancer diseases were twice as likely as patients dying due to cancer to be admitted to an ICU at the EOL. We also found that non-cancer patients may be more likely than cancer patients to die during an ICU admission. These findings add to the body of literature describing the substantial unwarranted variation in health care at the EOL. They also emphasize the need for further investigation into reasons behind this variation to enable provision of the optimal care for patients at the EOL, regardless of diagnosis.

## Key messages

- Patients dying as a result of non-cancer chronic diseases are two times likelier than patients dying due to cancer to be admitted to an ICU within 6 months before they die.
- Non-cancer patients may be likelier than cancer patients to die during an ICU admission.
- Substantial variation was found among the causes of death regarding admission to an ICU and treatment during ICU admission at the EOL.

## Additional files

Additional file 1:
**Cumulative prevalence of causes of death, 2005–2011 in Denmark.** ^†^Total = 377,410. All decedents were aged 18 years and older. ICD-10 diagnoses were used to define chronic diseases as underlying cause of death. Cancer, diabetes, dementia, ischemic heart disease, cerebrovascular disease, and chronic obstructive pulmonary disease were included in the study. All other causes of death were excluded. ^‡^Heart failure was defined according to the National Indicator Project – cancer, diabetes, dementia, ischemic heart disease, cerebrovascular disease, and chronic obstructive pulmonary disease, and others were defined according to the Danish Registry of Causes of Death. (PDF 72 kb) Additional file 2:
**Modification of Charlson comorbidity index by causes of death.** *If these diseases were present in patients as the underlying cause of death, as indicated by the shaded boxes, no points were added to the CCI score. For example, a person with myocardial infarction and ulcer disease as comorbidities who died as a result of ischemic heart disease will have a CCI score of myocardial infarction (1) + ulcer disease (1) = total CCI score (2) and points for underlying cause of death, ischemic heart disease (1) = total modified CCI score (1). ^†^These diseases were not present as underlying causes of death. (PDF 63 kb) Additional file 3:
**Model for aggressiveness of treatment during admission to the ICU.** Points assigned for each treatment modality. For example, mechanical ventilation (1), non-mechanical ventilation (0), and dialysis (1) = total score (2). Points grouped as 0–2 points: partial organ support treatment; 3 points: full organ support treatment. (PDF 42 kb)

## Abbreviations

aPR: Adjusted prevalence ratio
CCI: Charlson comorbidity index
CI: Confidence interval
CLF: Chronic liver failure
COPD: Chronic obstructive pulmonary disease
DID: Danish Intensive Care Database
EOL: End of life
GP: General practitioner
ICU: Intensive care unit
IHD: Ischaemic heart disease
IQR: Interquartile range
NIV: Non-invasive ventilation
RRT: Renal replacement therapy
