# Supplementary material for: Intensive care at the end of life in patients dying due to non-cancer chronic diseases versus cancer: a nationwide study in Denmark
Source: Crit Care. 2015 Nov 24;19:413. doi: 10.1186/s13054-015-1124-1 (PMC4657209; doi:10.1186/s13054-015-1124-1)
Supplement: Additional file 1: — Cumulative prevalence of causes of death, 2005–2011 in Denmark. †Total = 377,410. All decedents were aged 18 years and older. ICD-10 diagnoses were used to define chronic diseases as underlying cause of death. Cancer, diabetes, dementia, ischemic heart disease, cerebrovascular disease, and chronic obstructive pulmonary disease were included in the study. All other causes of death were excluded. ‡Heart failure was defined according to the National Indicator Project – cancer, diabetes, dementia, ischemic heart disease, cerebrovascular disease, and chronic obstructive pulmonary disease, and others were defined according to the Danish Registry of Causes of Death. (PDF 72 kb) [file 13054_2015_1124_MOESM1_ESM.pdf]

Additional file 1 Cumulated prevalence of causes of death 2005-2011 in Denmark†

	Deceased, number	(% of all decedents)	ICD-10 codes
Cancer	106,456	(28.2%)	C00-C97
Diabetes	9,150	(2.4%)	E10–E14
Dementia, incl. Alzheimer's	18,298	(4.8%)	F01, F03, G30
Ischemic heart disease	39,466	(10.5%)	I20-25
Heart failure‡	10,779	(2.9%)	I11.0, I13.0, I13.2, I42.0, I42.6, I42.7, I42.9, I50.0, I50.1, I50.9
Cerebrovascular disease	28,522	(7.6%)	I60-I69
Chronic obstructive pulmonary disease	22,120	(5.9%)	J41-J44, J47
Chronic liver disease	5,963	(1.6%)	K70, K73-74
Other	136,741	(36.2%)	
Infectious incl. parasitic diseases	5,554	(1.5%)	A00-B99
In situ neoplasms, benign neoplasms, neoplasms of uncertain or unknown behaviour	2,387	(0.6%)	D00-48
Diseases in the blood and blood forming organs, diseases comprising the immune system	1,736	(0.5%)	D50-89
Endocrine, nutritional, and metabolic diseases excluding diabetes	3,614	(1.0%)	E00-90 (without E10–E14)
Mental and behavioural disorders excluding dementia	7,047	(1.9%)	F01, F03-99 (without F01, F03)
Diseases of the nervous system and sensory organs	6,347	(1.7%)	G00-31, G35-H95 (without dementia G30)
Diseases of the heart	19,131	(5.1%)	I00-25, I27, I 30-51 (without ischemic heart disease I20-25, and heart failure I11.0, I13.0, I13.2, I42.0, I42.6, I42.7, I42.9, I50.0, I50.1, I50.9)
Other circulatory diseases	9,907	(2.6%)	I26, I28, I60-99 (without cerebrovascular disease I60-I69)
Diseases of the respiratory system	17,186	(4.6%)	J00-99 (without chronic obstructive pulmonary disease J41-J44, J47)
Diseases of the digestive system	13,110	(3.5%)	K00-99 (without chronic liver failure K70, K73-74)
Diseases of the skin and subcutaneous tissue	481	(0.1%)	L00-99
Diseases of the musculoskeletal system and connective tissue	2,439	(0.6%)	M00-99
Diseases of the	5,970	(1.6%)	N00-98

genitourinary system			
Complications pregnancy, childbirth, and the puerperium	15	(0.0%)	O00-99
Certain conditions originating in the perinatal period	19	(0.0%)	P00-96
Congenital malformations, deformations, and chromosomal abnormalities	562	(0.1%)	Q00-99
Symptoms, signs and abnormal clinical and laboratory findings, not elsewhere classified	13,357	(3.5%)	R00-98, R99.9
Accidents	10,810	(2.9%)	V01-X59, Y40-69, Y70-86, Y88
Suicide and suicide attempts	4196	(1.1%)	X60-84, Y840
Homicide, assault	279	(0.1%)	X85-99, Y00-09, Y87.1
Events of undetermined intent	899	(0.2%)	Y10-34, Y87.2, Y899
Legal interventions and operations of war (police, military, state of war)	13	(0.0%)	Y35-36, Y89.0-89.1
Death with no medical information	11,594	(3.1%)	R99.0
Missing information	7,269	(1.9%)	-

† Total N=377,410. All decedents were aged 18 and older. ICD-10 diagnoses were used to define chronic diseases as underlying cause of death. Cancer, diabetes, dementia, ischemic heart disease, cerebrovascular disease, and chronic obstructive pulmonary disease were included in the study. All other causes of death were excluded.

‡Heart failure was defined according to the National Indicator Project – Cancer, diabetes, dementia, ischemic heart disease, cerebrovascular disease, and chronic obstructive pulmonary disease, and other were defined according to the Danish Registry of Causes of Death.
