# Supplementary material for: Intensive care at the end of life in patients dying due to non-cancer chronic diseases versus cancer: a nationwide study in Denmark
Source: Crit Care. 2015 Nov 24;19:413. doi: 10.1186/s13054-015-1124-1 (PMC4657209; doi:10.1186/s13054-015-1124-1)
Supplement: Additional file 2: — Modification of Charlson comorbidity index by causes of death. *If these diseases were present in patients as the underlying cause of death, as indicated by the shaded boxes, no points were added to the CCI score. For example, a person with myocardial infarction and ulcer disease as comorbidities who died as a result of ischemic heart disease will have a CCI score of myocardial infarction (1) + ulcer disease (1) = total CCI score (2) and points for underlying cause of death, ischemic heart disease (1) = total modified CCI score (1). †These diseases were not present as underlying causes of death. (PDF 63 kb) [file 13054_2015_1124_MOESM2_ESM.pdf]

Additional file 2 Modification of Charlson Comorbidity Index by causes of death

	Cancer	*Diabetes*	*Dementia*	*Ischemic heart disease*	*Heart failure*	*Cerebrovascular disease*	*COPD*	*Chronic liver failure*
Myocardial infarction*				■				
Congestive heart failure*					■			
Peripheral vascular disease†								
Cerebrovascular disease*						■		
Dementia*			■					
Chronic pulmonary disease*							■	
Connective tissue disease†								
Ulcer disease†								
Mild liver disease*								■
Diabetes type 1*		■						
Diabetes type 2*						■		
Hemiplegia†								
Moderate-to-severe renal disease*		■						
Diabetes type 1 with end organ damage*	■							
Diabetes type 2 with end organ damage*	■							
Any tumour*	■							
Leukaemia*								■
Lymphoma	■							
Moderate-to-severe liver disease†								
Metastatic solid tumour†								
AIDS†								

*If these diseases were present in patients as underlying cause of death, as indicated by the shaded boxes, 0 points were added to the CCI score. Example: A person with myocardial infarction and ulcer disease as comorbidity, who died of ischemic heart disease will have a CCI score of myocardial infarction (1) + ulcer disease (1)=total CCI score (2) – points for underlying cause of death, ischemic heart disease(1)=total modified CCI score (1)

†These diseases were not present as underlying cause of death
