# Supplementary material for: Intensive care at the end of life in patients dying due to non-cancer chronic diseases versus cancer: a nationwide study in Denmark
Source: Crit Care. 2015 Nov 24;19:413. doi: 10.1186/s13054-015-1124-1 (PMC4657209; doi:10.1186/s13054-015-1124-1)
Supplement: Additional file 3: — Model for aggressiveness of treatment during admission to the ICU. Points assigned for each treatment modality. For example, mechanical ventilation (1), non-mechanical ventilation (0), and dialysis (1) = total score (2). Points grouped as 0–2 points: partial organ support treatment; 3 points: full organ support treatment. (PDF 42 kb) [file 13054_2015_1124_MOESM3_ESM.pdf]

Additional file 3 Model for aggressiveness of treatment during admission to ICU

Points assigned	*Treatment modality*
1	Mechanical ventilation and/or non-mechanical ventilation treatment
1	Inotropic and/or vasopressor treatment
1	Dialysis treatment

Points assigned for each treatment modality. Example: Mechanical ventilation (1), non-mechanical ventilation (0), and dialysis (1) = total score (2)

Points grouped as 0-2 points: Partial organ support treatment and 3 points: Full organ support treatment.
